# DRCNN: decomposing residual convolutional neural networks for time series forecasting

**DOI:** 10.1038/s41598-023-42815-6

**Published:** 2023-09-23

**Authors:** Yuzhen Zhu, Shaojie Luo, Di Huang, Weiyan Zheng, Fang Su, Beiping Hou

**Affiliations:** 1 School of Automation and Electrical Engineering, Zhejiang University of Science and Technology, Hangzhou, 310000 China; 2 Hangzhou Science and Technology Development Branch of Zhejiang Dayou Industrial Co.,Ltd., Hangzhou, 310000 China

**Keywords:** Engineering, Electrical and electronic engineering

## Abstract

Recent studies have shown great performance of Transformer-based models in long-term time series forecasting due to their ability in capturing long-term dependencies. However, Transformers have their limitations when training on small datasets because of their lack in necessary inductive bias for time series forecasting, and do not show significant benefits in short-time step forecasting as well as that in long-time step as the continuity of sequence is not focused on. In this paper, efficient designs in Transformers are reviewed and a design of decomposing residual convolution neural networks or DRCNN is proposed. The DRCNN method allows to utilize the continuity between data by decomposing data into residual and trend terms which are processed by a designed convolution block or DR-Block. DR-Block has its strength in extracting features by following the structural design of Transformers. In addition, by imitating the multi-head in Transformers, a Multi-head Sequence method is proposed such that the network is enabled to receive longer inputs and more accurate forecasts are obtained. The state-of-the-art performance of the presented model are demonstrated on several datasets.

## Introduction

**Figure 1:**
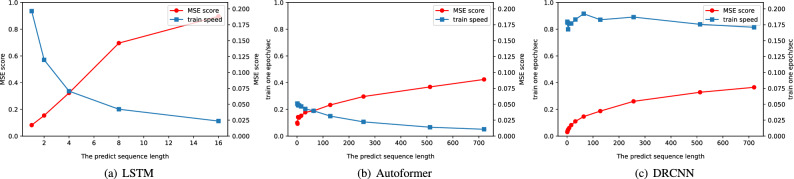
Model performance on ETTm2 in serveral output length.

With the success of neural networks in computer vision field and natural language processing^1, 2^, more and more neural network-based time series forecasting methods have been proposed^3, 4^, which are mainly divided into three categories, i.e. RNN-based methods, PredRNN^5^, DeepAR^6^. CNN-based methods WaveNet^7^, TCN^8^, SCINet^9^, Transformer-based methods Informer^10^, Autoformer^11^, FEDformer^12^. The RNN-based methods are autoregressive methods^13^, which mean that the predicted results of each step depend on previous results. When the length of the sequence to be predicted is short, the autoregressive method helps the model to achieve better results. However, when the length of the prediction sequence becomes longer, the autoregressive method leads to accumulation of errors. Meanwhile the time cost of the model increases linearly. Therefore, RNN-based methods are difficult to be applied to long-term series prediction. For example, Fig. 1 shown that LSTM^14^, an excellent RNN network, has small MSE scores in predictions at short time steps and is also fast. But when the time step becomes longer, the MSE score rises with a large gradient and the speed becomes slow.

Different from RNNs which usually use rolling prediction, Transformer-based methods output all predictions simultaneously^10^. This one-shot approach prevents the model from accumulating errors due to longer predictions^15^. However, we find Transformers have shortcomings too. Modeling of sequence in Transformers^16^ are based on self-attention mechanism which uses an attention score matrix to correlate importance among sequences. Thus, Transformers can merely learn relative relationship of sequence, rather than from the context of sequence. Therefore, Transformer uses an extra positional encoding to learn this context, which does not actually change the absence of Transformer’s inductive bias^17^. In natural language processing, Transformers can be trained with billions of characters, such huge dataset helps Transformers to train well. But in general time series forecasting tasks, we usually only have tens of thousands of pieces of data, because collecting data is expensive in time series forecasting. For example, the ETT dataset^10^ was collected from power transformers, and a subset of it, ETT-small-m1, was sampled at 15-min intervals, yielding 70,080 data points over two years of collection. Although many approaches have been proposed to improve self-attention by introducing frequency domain analysis, they do not fully address the shortcomings of Transformer’s inability to take advantage of contextual learning. Because of such advantage, the Transformer-based model performs poorly in short time series forecasting, in which model needs the ability to better incorporate context. We tested the performance of Autoformer when predicting outputs of different lengths in Fig. 1 which shows that the MSE score of Autoformer is greatly improved compared to RNN network in long-term series prediction, but RNN is better than Autoformer in short-term series prediction. In terms of training speed, Autoformer training speed is slower, but the speed does not significantly change as the length of the predicted sequence vary.

CNN-based methods convolve the input sequence with a certain step size through a convolution kernel. This method itself implies a hypothesis that adjacent data have greater correlation, which happens to be the inductive bias that Transformers lack. At the same time, unlike RNN-based methods, CNNs can easily adopt one-shot methods to predict like Transformers.

CNNs are widely used in time series analysis of sensor data^18, 19^, because they have strength in recognizing simple patterns in data and then using these simple patterns to generate more complex patterns in higher-level layers count data or signal data with a fixed length period. We also see that CNN-based methods achieve SOTA performance in time series forecasting^9^, and based on the above considerations, we believe that the excellent performance of CNNs can be extended to long-term time series forecasting, allowing the model achieves good performance in both short-term and long-term time series forecasting. We believe that CNN-based methods will be an excellent solution for time series forecasting.

Based on the above discussion, a DRCNN is proposed. By adopting the efficient design of Transformer-based methods, the model can achieve better results when dealing with long-term time series forecasting, and we also design modules that allow the model to efficiently use longer inputs and have better performance. Our main contributions are as follows:

- We propose a Multi-head Sequence operation. By downsampling the sequence into multiple subsequences, the number of subsequences is used as a hyperparameter, each subsequence extracts features independently and concat them at the end. The model can use more information from longer input sequences to obtain better results and this parallelized processing can greatly improve the speed of the model. We have also extended this approach to Transformer to obtain performance improvements.
- We design the DR-Block. DR-Block combines the method proposed above and uses the structure of AddNorm to improve the performance of the convolutional layer. It can effectively capture long-term and short-term time series information, so that the model can achieve good results in both long-term and short-term prediction.
- We design a structure to decompose the input sequence into trend terms and residual terms at multiple scales, each using a convolution block to learn the features of the corresponding scale, and the sum of the learned features is the output of the model. This design allows the model to make better use of the input sequence. the corresponding scale, and the sum of the learned features is the output of the model. This design allows the model to make better use of the input sequence

Extensive experiments show that our model can achieve SOTA results by utilizing longer input sequence and gain better result. We tested our model on seven datasets, all achieved SOTA results. In particular, for the ETT dataset, a relative improvement of more than 10% in terms of mean squared error is achieved compared to SOTA methods. We briefly show the performance of the model in Fig. 1. DRCNN has better short-term prediction performance than RNN, and the training time does not change much with the increase of the prediction sequence.

## Related work

Classical time series forecasting methods, such as VAR^20^ and ARIMA^21^ are indispensable part in the field of time series forecasting. However, these models are constrained by linearity assumptions and the number of covariates. As a result, it’s difficult to handle complex nonlinear time series for them^22^.

RNN-based methods memorize previous information and use the previous information to influence the output of subsequent nodes. In this way, the model can perform well on short-term series predictions, but the way the output is generated in multiple steps makes the model perform poorly on long-term series. There are many works to improve this weaknesses by introducing other methods. LSTNet^23^ introduces a CNN to capture short and long-term temporal patterns. DARNN^24^ introduces attention to resolve long-term dependencies of predictions. The introduction of Transformer makes long-term sequential prediction possible. By using the attention scoring matrix, the model can well resolve the long-term dependence of the input sequence, thus improving the accuracy of long-time time series prediction. A lot of work also builds on it to improve the performance of the model again. Informer^10^ designs a decoder that outputs all predicted sequences at one time, avoiding accumulated errors. TCCT^25^ improves the performance of the model on Informer using a convolutional structure parallel to self-attention, and this improvement gives us confidence in the power of convolution for time series prediction. The sequence decomposition block proposed by Autoformer^11^ greatly improves the performance of the model by decomposing the input into residual terms and trend terms for prediction respectively, and at the same time improves the attention mechanism by using Fast Fourier Transform, and proposes an autocorrelation mechanism. Our model also uses the sequence decomposition block to decompose the input. Fedformer^12^ design a more complete frequency domain analysis method on it, which further improved the performance of the model. Our model structure is inspired by the mixture of experts method in FEDformer. People also see the potential of CNNs for time series forecasting. Using a CNN-derived TCN^8, 26^ structure easily outperforms RNNs on many tasks^9^. TCN uses dilated convolution and grouped convolution to improve the performance of the model, while the author uses causal convolution to prevent future information leakage, which is considered to limit the performance of the model in SCINet^9^, as such way actually reduces historical information. SCINet adopts a one-shot output method while abandoning the design of causal convolution. In addition, SCINet designs a binary downsampling method so that the model can learn more refined features. Our DR-Block is inspired by Transformer and introduces both grouped convolution and dilated convolution, which are proven in several experiments.

## Methods

### Problem definition

Time series forecasting methods try to predict future time series from previous time series. In particular, given a previous time series $X_{t_1:t_2}=\{x_{t_1},x_{t_1+1},...,x_{t_2}\}$, each variable *x* with $d_x$ dimension, and we should predict $X_{t_3:t_4}=\{x_{t_3},x_{t_3+1},...,x_{t_4}\}$.

Therefore, the crux of the problem lies in effectively mapping a two-dimensional matrix onto another two-dimensional matrix, where the former contains the time dimension and the feature dimension. This perspective unifies two distinct forecasting scenarios: multivariate time-series forecasting and univariate time-series forecasting. The difference lies in the number of feature dimensions they encompass. In our proposed approach, we initially extract the feature vectors of the time series through the model, subsequently aligning the feature vectors with their corresponding outputs via the utilization of two linear layers.

In this section, we focus our methods on time series forecasting due to their superior performance, especially for multivariate time series.

### Multi-head sequence method

Inspired by Transformer’s multi-head structure, we designed Sequence multi-head by downsampling the sequence into multiple subsequences, we can able to obtain multiple representations of the original sequence, which facilitates the model to get the patterns for the sequence. The experimental results show that how this approach affects the optimal input sequence length of the model and improve the performance.

**Figure 2:**
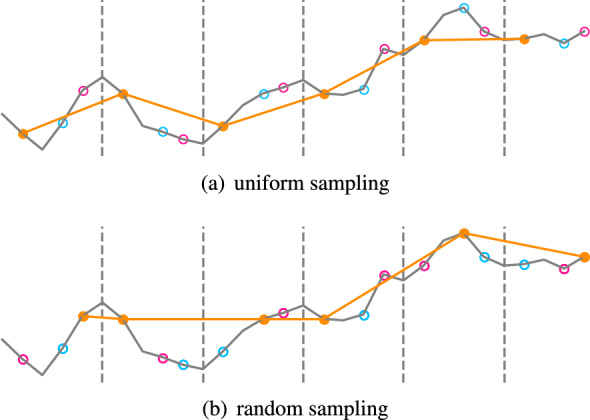
Two different ways to sampling.

We tried two ways to down sample. One way is to use uniform sampling. Uniform sampling means that the original sequence is evenly divided into subsequence segments, and each segment of the original sequence is sampled in a certain order. For instance, the original sequence in Fig. 2a is divided into 6 sub-segments for each field we sample in the same order.

Another way is random sampling. In order to avoid the loss of information, we will ensure that every value of the original sequence is sampled, so we divide the original sequence first in the same way as uniform sampling. For each segment, we choose a value by random sampling, but make sure that every point is sampled. For example, in Fig. 2b, the orange line is generated by this random sampling method. These two approaches behave differently on different datasets. Through our tests, we found that uniform sampling performs better on smoother sequences, while random sampling is better on sequences with strong noise.

Both of them can be implemented with simple code, and the pseudo-code is given below. For uniform sampling, the $k{\text {th}}$ subsequence *S*[*k*] :

1 $$\begin{aligned} S[k] = X[:, k{:}{:}N, :]. \end{aligned}$$

N means the total number of subsequences.

For random sampling, we need to generate a sampling matrix:

2 $$\begin{aligned} IDX[k] = randperm(N) + k \times N. \end{aligned}$$

*randperm* is a function that generates a random permutation of integers within a given range. and the sampled subsequence *S*[*k*] :

3 $$\begin{aligned} S[k] = X[:, IDX[k], :]. \end{aligned}$$

**Figure 3:**
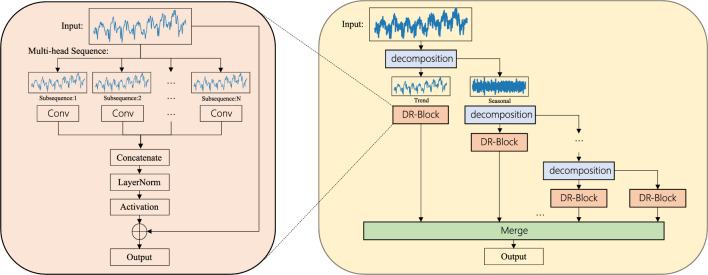
Structure of DRCNN.

We also attempt to illustrate why MS has positive implications for time series forecasting from the perspective of serial smoothing. The method of smoothing series is widely used in traditional time series forecasting. For example, ARIMA^21, 27^ uses moving average to smoothing the sequence and it has proved effective. Autoformer’s attempt to introduce moving averages led us realize that perhaps smooth sequences are applicable in emerging deep learning-based methods. The subsequence generate by downsampling can be regarded as a smoothed sequence. Each subsequence has a different representation while the pattern is similar. Each convolution block can learn the encoded representation information in different subspaces which enhances the expressiveness of the model.

### DR-block

With the MS introduced above, we designed a convolutional module or DR-Block, which as follows:

4 $$\begin{aligned} &S = Sample(X) \\&Concat = Concatenate(Conv_1(S[1]),...,Conv_N(S[N])) \\&DRBlock(X) = X + Activation(LayerNorm(Concat)). \end{aligned}$$

*Sample* here refers to the two sampling methods mentioned above, and *S* is a series of subsequences obtained after sampling. These subsequences are concatenated together after extracting features through a series of convolutional layers independent of the parameters as the intermediate variable *Concat*. *Concat* is added to the original input *X* as the output of DRBlock after passing the *LayerNorm* and activation function which is GELU^28^ in our implementation.

LayerNorm (Layer Normalization) is a technique used in deep learning to normalize the activations of a neural network layer. It helps stabilize the training process and improve the convergence of the model.

5 $$\begin{aligned} &LayerNorm(X) = \frac{1}{\sqrt{\sigma ^2 + \varepsilon }} \cdot (X - \mu ) \\&\mu = \text {mean}(X)\text {(Mean of the input)} \\&\sigma = \text {std}(X)\text {(Standard deviation of the input)} \\&\varepsilon = \text {small constant}. \end{aligned}$$

LayerNorm is applied element-wise to each dimension of the input tensor *X*. It normalizes the values by subtracting the mean and dividing by the standard deviation. The scaling factor ensures that the normalized values have the desired variance.

The design of DR-Block follows the design of AddNorm^29, 30^ in Transformer, as shown in Fig. 3, we will pass the input through the convolution layer and activate it, we also use Dropout^31^ and norm to prevent overfitting, and finally add it to the original input as the output. AddNorm is the key for Transformer to build an effective deep architecture^32^. This also helps the decomposition structure introduced in the next section to be consistently effective.

**Table 1:** Multivariate time-series forecasting results on six datasets.

Methods		DRCNN		FEDformer		Autoformer		Informer		Reformer		Transformer	
Metric		MSE	MAE	MSE	MAE	MSE	MAE	MSE	MAE	MSE	MAE	MSE	MAE
ETTm2	24	**0.092**	**0.190**	0.115	0.226	0.153	0.259	0.318	0.423	0.263	0.371	0.179	0.309
	96	**0.164**	**0.252**	0.203	0.287	0.255	0.339	0.365	0.453	0.658	0.619	0.735	0.626
	192	**0.223**	**0.294**	0.269	0.328	0.281	0.340	0.533	0.563	1.078	0.827	1.185	0.854
	336	**0.273**	**0.331**	0.325	0.366	0.339	0.372	1.363	0.887	1.549	0.972	1.293	0.857
	720	**0.358**	**0.393**	0.421	0.415	0.422	0.419	3.379	1.338	2.631	1.242	2.404	1.132
Electricity	24	**0.102**	**0.199**	0.164	0.284	0.163	0.284	0.265	0.357	0.280	0.376	0.224	0.332
	96	**0.132**	**0.229**	0.183	0.297	0.201	0.317	0.274	0.368	0.312	0.402	0.263	0.361
	192	**0.143**	**0.242**	0.195	0.308	0.222	0.334	0.296	0.386	0.348	0.433	0.278	0.369
	336	**0.161**	**0.260**	0.212	0.313	0.231	0.338	0.300	0.394	0.350	0.433	0.278	0.369
	720	**0.190**	**0.288**	0.231	0.343	0.254	0.361	0.373	0.439	0.340	0.420	0.292	0.375
Exchange	24	**0.023**	**0.100**	0.054	0.168	0.092	0.228	0.498	0.547	0.656	0.626	0.278	0.409
	96	**0.078**	**0.199**	0.139	0.276	0.197	0.323	0.847	0.752	1.065	0.829	0.589	0.636
	192	**0.160**	**0.292**	0.256	0.369	0.300	0.369	1.204	0.895	1.188	0.906	1.656	1.030
	336	**0.293**	**0.408**	0.426	0.464	0.509	0.524	1.672	1.036	1.357	0.976	1.409	0.986
	720	**0.636**	**0.610**	1.090	0.800	1.447	0.941	2.478	1.310	1.510	1.016	1.615	1.054
Traffic	24	**0.354**	**0.257**	0.550	0.359	0.609	0.384	0.762	0.435	0.727	0.412	0.770	0.457
	96	**0.381**	**0.269**	0.562	0.349	0.613	0.388	0.719	0.391	0.732	0.423	0.703	0.395
	192	**0.388**	**0.270**	0.562	0.346	0.616	0.382	0.696	0.379	0.733	0.420	0.744	0.413
	336	**0.404**	**0.280**	0.570	0.323	0.622	0.337	0.777	0.420	0.742	0.420	0.684	0.373
	720	**0.450**	**0.306**	0.596	0.368	0.660	0.408	0.864	0.472	0.755	0.423	0.674	0.367
Weather	24	**0.101**	**0.131**	0.152	0.236	0.218	0.318	0.271	0.337	0.655	0.583	0.170	0.254
	96	**0.165**	**0.213**	0.217	0.296	0.266	0.336	0.300	0.384	0.689	0.596	0.438	0.445
	192	**0.210**	**0.253**	0.276	0.336	0.307	0.367	0.598	0.544	0.752	0.638	0.593	0.367
	336	**0.265**	**0.291**	0.339	0.380	0.359	0.395	0.578	0.523	0.639	0.596	0.444	0.474
	720	**0.332**	**0.340**	0.403	0.428	0.578	0.578	1.059	0.741	1.130	0.792	0.654	0.579
ILI	24	**2.141**	1.004	2.203	**0.963**	3.483	1.287	5.764	1.677	4.400	1.382	4.138	1.329
	36	**2.222**	1.042	2.272	**0.976**	3.103	1.148	4.755	1.467	4.783	1.448	4.430	1.423
	48	**2.178**	1.031	2.209	**0.981**	2.669	1.085	4.763	1.469	4.832	1.465	4.883	1.477
	60	**2.340**	1.084	2.545	**1.061**	2.770	1.125	5.264	1.564	4.882	1.483	5.202	1.571

Best-performing model values are in bold.

### DRCNN

Inspired by MOE Decomp in FEDformer which use a set of filters with different sizes to extract multiple trend components from the input signal and combining them as the final trend, we use a multi-resolution moving average to learn the features of the residual terms. Specifically, we choose a set of average ensemble layers with different convolutional kernel sizes. The kernels are sized from large to small, so that the trend terms decomposed from the residual terms will have multi-scale features. After obtaining the multi-scale trend terms and the final residual terms, we learn the importance of each term by MLP^33^, and the importance will be turned into coefficients between 0 and 1 by softmax, these coefficients will be multiplied with the corresponding terms respectively, these results will be summed up for the model.

For the outputs *Y* after convolutional layers, we use MLP to estimate their confidence levels separately and then use softmax to scale these confidence levels to a coefficient *I* that sums to *I*:

6 $$\begin{aligned} I = S(MLP(Y)), S_i(X) = \frac{e^{x_i}}{\sum _{j=1}^{n}e^{x_j}}. \end{aligned}$$

*S* here refers to the softmax function, which is a widely used activation function in neural networks for mapping the outputs of multiple neurons, into the (0,1) interval.

The final output is the sum of the dot products of *I* and *Y*:

7 $$\begin{aligned} Output = \sum _{i=0}^{n+1} I_i Y_i. \end{aligned}$$

DRCNN is based on DR-Block to obtain further performance improvement by decomposing the residuals. As shown in Fig. 3, we decompose the input of DRCNN into trend and residual terms by decomposition module. The decomposition module obtains the trend term by moving average and subtracts the input sequence from the trend term to obtain the residual term. We use a moving average with a larger window at the shallow level of DRCNN to obtain the trend term at the shallow level, and we use a smaller moving evaluation at the deep level to obtain the trend term at the deeper level.

We will use DR-Block to learn the features of the trend term obtained from the residual term. And the last remaining residual term will also use DR-Block to learn features which will be mapped to the length of the output sequence by linear after summing. This structure is also the factor that the model can utilize longer input sequences to obtain better performance. The above process can be described as:

8 $$\begin{aligned} &X^{trend}_k = SeriesDecomp_k(X_{k - 1}^{seasonal})\\&X_{k}^{seasonal} = X_{k - 1}^{seasonal} - X_{k}^{trend}\\&Y_k = DRBlock_k(X_{k}^{trend})\\&Y_{n + 1} = DRBlock_{n + 1}(X_{n}^{seasonal}), \end{aligned}$$

where $X_k^{trend}$ is obtained by decomposing the k-1th residual term by the kth *SeriesDecomp*. The kth residual term $X_k^{seasonal}$ is then derived from the $X_{k-1}^{seasonal}$ subtracted from $X_k^{trend}$. $Y_k$ is obtained by passing a $X_k^{trend}$ through the kth DRBlock, and $Y_{n+1}$ is obtained by passing the last residual term through the DRBlock. The *Y* obtained here will be calculated by the method above to produce the output.

*SeriesDecomp* utilizes the Autoformer idea, especially the following two processes:

9 $$\begin{aligned} SeriesDecomp(X) = AvgPool(Padding(X)). \end{aligned}$$

*AvgPool* acts as a moving average here, and *Padding* ensures that the length of the sequence does not change, so that the result of *SeriesDecomp* are used as trend term.

### Loss function

Typically, time series forecasting is optimized by either mean squared errors (MSE) or mean absolute errors (MAE) as the loss function, both of which are also used as evaluation metrics to determine how good the model is. We choose to use the SmoothL1 loss function to optimize the model. The SmoothL1 loss function can be written as:

10 $$\begin{aligned} smooth_{L1}(x) = {\left\{ \begin{array}{ll} 0.5x^2&{} \text {if x <1} \\ |x|-0.5&{} \text {otherwise} \end{array}\right. }. \end{aligned}$$

SmoothL1 loss is proposed in Fast RCNN^34^ and is widely used in target detection due to its excellent robustness. In the task of time series prediction, when the prediction value is too different from the ground truth, SmoothL1 loss ensures that the gradient is not too large to cause training instability, and when the prediction value is very small from the ground truth, it ensures that the gradient value is not so large that it destroys the network parameters. We find that there are benefits of introducing SmoothL1 loss in time series forecasting.

## Experiments

In this section, we present the experimental results of the above methods. We analyze these methods and explore them one by one, and try to illustrate how they affect performances.

**Table 2:** Univariate time-series forecasting results on three datasets.

Methods		DRCNN		FEDformer		Autoformer		Informer		Reformer		Transformer	
Metric		MSE	MAE	MSE	MAE	MSE	MAE	MSE	MAE	MSE	MAE	MSE	MAE
ETTm2	24	**0.019**	**0.092**	0.021	0.105	0.028	0.117	0.069	0.207	0.042	0.150	0.029	0.114
	96	**0.061**	**0.182**	0.063	0.189	0.065	0.189	0.080	0.217	0.131	0.288	0.088	0.235
	192	**0.093**	**0.231**	0.102	0.245	0.118	0.256	0.112	0.259	0.195	0.360	0.124	0.269
	336	**0.124**	**0.270**	0.130	0.279	0.154	0.305	0.166	0.314	0.220	0.381	0.163	0.315
	720	**0.163**	**0.317**	0.178	0.325	0.182	0.335	0.228	0.380	0.290	0.442	0.223	0.379
Electricity	24	**0.130**	**0.257**	0.233	0.365	0.379	0.473	0.265	0.357	0.213	0.340	0.207	0.338
	96	**0.218**	**0.325**	0.253	0.370	0.341	0.438	0.258	0.367	0.275	0.379	0.311	0.402
	192	**0.268**	**0.359**	0.282	0.386	0.345	0.428	0.285	0.388	0.304	0.304	0.313	0.406
	336	**0.287**	**0.379**	0.346	0.431	0.406	0.470	0.336	0.423	0.370	0.448	0.455	0.495
	720	**0.363**	**0.441**	0.422	0.484	0.565	0.581	0.607	0.599	0.460	0.511	0.498	0.529
Exchange	24	**0.025**	**0.121**	0.037	0.149	0.092	0.228	0.498	0.547	0.095	0.243	0.050	0.179
	96	**0.090**	**0.233**	0.131	0.284	0.241	0.387	1.327	0.944	0.298	0.444	0.283	0.409
	192	**0.183**	**0.333**	0.277	0.420	0.300	0.369	1.258	0.924	0.777	0.719	1.912	1.001
	336	**0.324**	**0.451**	0.426	0.511	0.509	0.524	2.179	1.296	1.833	1.128	2.339	1.179
	720	**0.852**	**0.715**	1.162	0.832	1.260	0.867	1.280	0.953	1.203	0.956	1.035	0.772

Best-performing model values are in bold.

### Dataset

#### ETT (electricity transformer temperature)^10^

The ETT dataset was collected from two counties in China over 2-year period. By varying the sampling interval, the ETT is divided into four datasets ETTh1, ETTh2, ETTm1, and ETTm2. 1 or 2 indicates the different counties from which the data originate. h represents a 1-h sampling interval and m represents a 15-min sampling interval. Specifically, ETTh1 and ETTh2 contain 17,420 data points and ETTm1 and ETTm2 contain 69,680 data points. These datasets consist of the same 8 features.

#### ECL (electricity consumption load)

The ECL consists of the hourly electricity consumption of 321 users over a 2-year period and contains 26,304 data points.

#### Exchange^35^

Exchange records daily exchange rates for eight different countries from 1990 to 2016 and contains 7588 data points.

#### Weather

The dataset records 21 climatic characteristics of Thuringia in 2020, including temperature, humidity and barometric pressure, with a sampling period of 10 min and a total of 52,696 data points.

#### Traffic

The traffic dataset is roadway occupancy recorded by 861 sensors on San Francisco Bay Area freeways, with a data sampling period of 1 h for a total of 17,544 data points.

#### ILI (influenza-like illness)

This dataset records data on patients with influenza-like illness recorded by the Centers for Disease Control between 2002 and 2021, and is specific to the proportion of patients with ILI out of the total number of patients. The data sampling period was one week totaling 966 data points.

All datasets were processed by normalization prior to training, and we employed the widely used dataset processor of Autoformer (https://github.com/thuml/Autoformer). All compared models were tested under the same data division.

### Setup

#### Baselines

In order to compare with our proposed DRCNN, we chose five SOTA Transformer models namely FEDformer, Autoformer, Informer, Reformer^36^, Transformer. We place the comparison of DRCNN with other CNN-based networks in other studies. Because the original authors of these Transformer-based models tested more datasets and more output length.

#### Implementation details

We use the ADAM^37^ optimizer to optimize the parameters and a learning rate decay^38^ of 0.5 to train our model. We also utilize an early stop training strategy to avoid overfitting. The training epochs were set to 10 and the batch size was set to 128. All experiments were done on PyTorch^39^ and trained on a single NVIDIA RTX3090 24GB GPU. For the fairness of the comparison, we used a standard setup, for the ETT dataset we divided it into 6:2:2, for the other datasets we used a ratio of 7:1:2 and divided all datasets into training, validation and test sets in chronological order.

#### Metrics

We employ MSE and MAE as our evaluation metrics, which as follows:

11 $$\begin{aligned} &MSE = \frac{1}{n} \sum _{i=1}^{n}(\hat{y_i} - y_i)^2,\\&MAE = \frac{1}{n} \sum _{i=1}^{n}|\hat{y_i} - y_i|. \end{aligned}$$

$\hat{y}$ refers here to the predictions of the model, and y is used as the ground truth. The reason why we use MAE and MSE as evaluation metrics is that the two evaluation metrics can be used to evaluate the performances of our model by comparing the corresponding evaluation metrics. Although metrics with scale consistency such as MAPE provide a better understanding of the relative percentage for error measurements. MSE and MAE still have some advantages, such as the fact that both evaluation metrics provide a visual representation of the prediction accuracy. Besides real-world time series often have outlier values that are not due to measurement error. MSE and MAE can reflect prediction errors if there are large deviations or outliers in the dataset.

### Main result

To better demonstrate the effectiveness of DRCNN on time series forecasting, we performed multivariate time series forecasting and univariate time series forecasting on several datasets. Multivariate time series forecasting requires forecasting all input features, while univariate time series forecasting forecasts a specific feature. For the ILI dataset our forecasting horizon $T\in \{24,36,48,60\}$ because this dataset is small with only a few thousand data. For the others our forecasting horizon $T\in \{24,96,192,336,720\}$.

For the Transformer-based models we used 24 inputs on the ILI and the rest used 96 inputs, which is the setup for these models to achieve SOTA scores. For DRCNN, we used 96-length inputs on the ILI and Exchange-rate datasets, and 720-length inputs for the other datasets, which helped the models to achieve excellent performance on each dataset. We choose MSE and MAE as evaluation metrics, and a smaller evaluation metric means better performance.

#### Multivariate time-series forecasting

In Table 1, DRCNN achieves an absolute advantage. We get 18% MSE improvement on the ETTm2 dataset, 27.8% MSE improvement on the electricity dataset, 42.3% MSE improvement on the foreign exchange dataset, 30.4% MSE improvement on the traffic dataset, 24.1% MSE improvement on the weather dataset, and 2% MSE improvement on the ILI dataset.

We speculate that the reason for only a small improvement on the ILI dataset is that the dataset is too small, with only 960 data, and the sampling period is too long for the sequences to be smooth enough for our proposed MS to work. For all other data sets, our model achieves SOTA results, obtaining an average improvement of more than 20% compared to previous models. On short time series, i.e., 24-length outputs, we obtain even greater performance improvements, For example, on the power dataset, we have a 37.8% improvement for 24-length outputs, compared to an average of 27.8% for this dataset. This may indicate that these Transformer-based models focus too much on the prediction of long time series and not very well on the prediction of short time series, and the use of Transformer-based models for the prediction of short time series may be a very interesting topic to explore.

#### Univariate time-series forecasting

As shown in Table 2, we tested the univariate time series prediction results of DRCNN on three datasets. The parameter settings were consistent with multivariate time series prediction, with only the feature input dimension modified. DRCNN achieves a 6.1% MSE improvement on ETTm2. Besides, DRCNN achieved an 18.8% MSE improvement on the Electricity dataset and a 29.6% improvement on the Exchange dataset.

### Ablation studies

All our ablation experiments are performed on the ETTm2 prediction 720 length output, which is a good task to test the performance of the model on long-term series prediction, and a good form of performance to verify the effectiveness of the proposed method above.

#### Ablation study on input length

**Figure 4:**
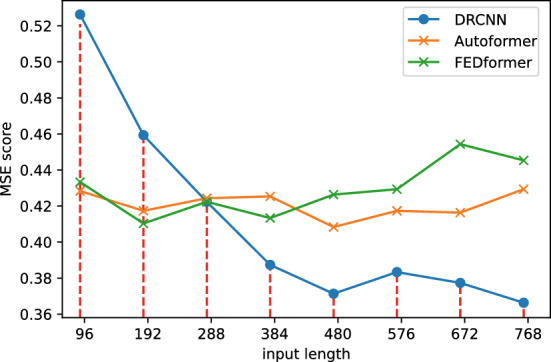
Trend of model performance with increasing input length.

In justifying our choice of utilizing 720-length inputs for our main experiments, while employing 96-length inputs for other baseline models, we believe such comparisons are meaningful. An important aspect of this paper is to investigate the potential for achieving improved prediction results by utilizing longer input sequences, a challenge that other models may not readily address. Our experiments further underline this perspective. As we gradually increased the input length beyond 96, the performance of the other models, as depicted in Fig. 4, exhibited a decline in performance.

**Figure 5:**
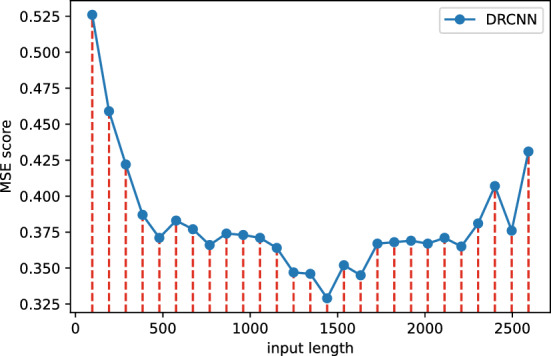
More complete input length settings in DRCNN.

We find that the performance still improves after the input length is beyond 1000, until the model performance drops back and is basically stable when the input length approaches 2000, which was shown in Fig. 5.

#### Ablation study on multi-head sequence

**Figure 6:**
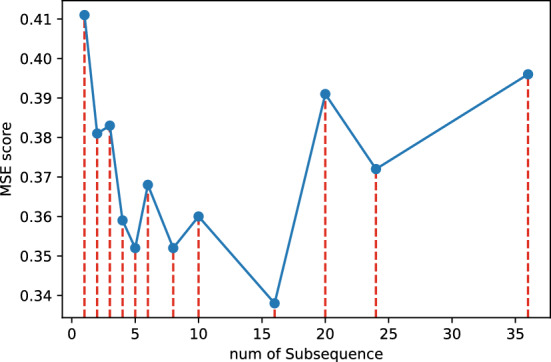
Trend of model performance with more subsequence.

In Fig. 6, our experiments demonstrate that as the number of subsequences grows, the MSE error of the model first decreases significantly and then will rebound, which is in line with expectations. This observation underscores the significance of choosing an appropriate number of subsequences. It enables the model to effectively capture sequence features and harness input from long sequences. However, it also highlights the need to strike a balance, as an excessive degree of subsequences can potentially disrupt timing information and result in a decline in model performance.

#### Ablation study on network layers

**Figure 7:**
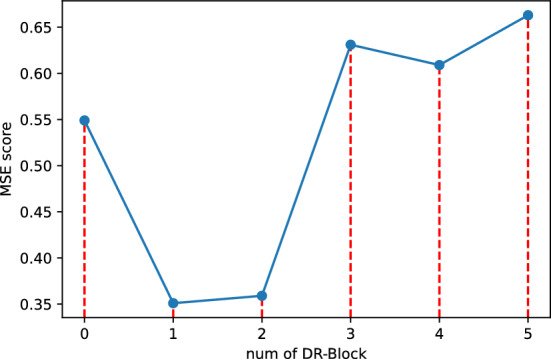
Trend of model performance with more DR-block.

Figure 7 shows the trend of network performance as the number of layers increases, and the number of subsequences is set to 5. Experimentally, it is shown that a network with 1 to 2 layers can extract the input features well, and a network that is too deep can lead to overfitting and make the model perform worse.

#### Ablation study on sampling

The two methods we propose to obtain multi-headed subsequences have their own advantages and disadvantages on different datasets. We tested the performance of the two methods on the two datasets. The experimental results are shown in Table 3.

**Table 3:** Uniform sampling vs. random sampling.

Methods		Uniform		Random	
Metric		MSE	MAE	MSE	MAE
ETTm2	24	**0.103**	**0.204**	0.112	0.215
	96	**0.178**	**0.269**	0.176	0.271
	192	**0.243**	**0.314**	0.246	0.318
	336	**0.294**	**0.349**	0.332	0.357
	720	0.375	0.399	**0.361**	**0.387**
Traffic	24	0.359	**0.254**	**0.356**	**0.254**
	96	0.393	**0.269**	**0.389**	**0.269**
	192	0.404	0.274	**0.399**	**0.274**
	336	0.421	0.283	**0.416**	**0.282**
	720	0.471	0.306	**0.465**	**0.306**

Best-performing model values are in bold.

#### Ablation study on loss function

Table 4 shows the results obtained by using SmoothL1 MSE and MAE as loss functions on ETTm2. Using MSE or MAE directly as the objective function is not as effective as using the SmoothL1 function indirectly.

**Table 4:** SmoothL1 vs. MSE.

Loss		SmoothL1		MSE		MAE	
Metric		MSE	MAE	MSE	MAE	MSE	MAE
ETTm2	24	**0.092**	**0.190**	0.104	0.209	0.101	0.202
	96	**0.164**	**0.252**	0.179	0.274	0.168	0.257
	192	**0.223**	**0.294**	0.243	0.318	0.230	0.298
	336	**0.273**	**0.331**	0.301	0.358	0.284	0.335
	720	**0.358**	0.393	0.366	0.407	0.372	**0.386**

Best-performing model values are in bold.

#### Other studies

In Table 5, we tried using our proposed MS on the Transformer while retaining the original feature dimension Multi-heads, and our proposed MS mechanism helped the original Transformer to achieve an 18% performance improvement. However, the simple application of MS in Transformer has led us to believe that this approach can be extended to other Transformer-based models as well. We believe that this is a good direction to improve the Transformer-based model.

**Table 5:** Transformer with MS.

Methods		Transformer*		Transformer	
Metric		MSE	MAE	MSE	MAE
ETTm2	24	0.201	0.327	**0.179**	**0.309**
	96	**0.473**	**0.520**	0.735	0.626
	192	**0.911**	**0.713**	1.185	0.854
	336	**1.211**	**0.831**	1.293	0.857
	720	**2.239**	**1.109**	2.404	1.132

Best-performing model values are in bold.

Beyond the initial time we also did comparison experiments with SCINet and ARIMA. Because these models compared different datasets and different output lengths, we finally decided to put their results in here, and we also obtained SOTA results in the comparison with these models. As shown in Table 6.

**Table 6:** Comparison with SCINet and ARIMA.

Methods		DRCNN		SCINet		ARIMA	
Metric		MSE	MAE	MSE	MAE	MSE	MAE
ETTh1	24	**0.313**	**0.364**	0.341	0.379	0.108	0.284
	48	**0.340**	**0.379**	0.368	0.395	0.175	0.424
	168	**0.373**	**0.404**	0.451	0.457	0.396	0.504
	336	**0.459**	**0.464**	0.502	0.497	0.468	0.593
	720	**0.493**	**0.506**	0.583	0.560	0.659	0.766
ETTh2	24	**0.166**	**0.265**	0.188	0.288	3.554	0.445
	48	**0.222**	**0.307**	0.279	0.358	3.190	0.474
	168	**0.405**	**0.436**	0.505	0.504	2.800	0.595
	336	**0.518**	**0.504**	0.618	0.560	2.753	0.738
	720	**0.898**	**0.685**	1.074	0.761	2.878	1.044
ETTm1	24	0.199	0.279	**0.126**	**0.229**	0.090	0.206
	48	0.263	0.323	**0.169**	**0.274**	0.179	0.306
	96	0.295	0.345	**0.191**	**0.291**	0.272	0.399
	288	**0.349**	**0.379**	0.365	0.415	0.462	0.558
	672	**0.408**	**0.414**	0.713	0.604	0.639	0.697

Best-performing model values are in bold.

## Conclusion

In this paper, we proposed a CNN-based model called DRCNN. In DRCNN, a Multi-head Sequence method is introduced to divide the sequence into multiple subsequences by downsampling the input sequence. This approach helps the model make predictions using information from longer input sequences. Based on this, we design a convolution block, which can effectively extract temporal features through the method of Multi-head Sequence and the effective structure of AddNorm. We also use cyclic decomposition to smooth the sequence to help the model achieve good results. DRCNN achieves SOTA prediction performance in various experimental settings on different datasets, which demonstrates the effectiveness of our DRCNN.

## Data Availability

The ETT dataset is available at https://github.com/zhouhaoyi/ETDataset. The ECL dataset is available at https://archive.ics.uci.edu/ml/datasets/ElectricityLoadDiagrams20112014. The Exchange dataset is available at https://github.com/laiguokun/ multivariatetime-series-data. The Weather datasetis available at https://www.bgcjena.mpg.de/wetter/. The Traffic dataset is available at http://pems.dot.ca.gov. The ILI dataset is available at https://gis.cdc.gov/grasp/fluview/fluportaldashboard.html.

## Code availability

The code is available at https://github.com/acrndr/DRCNN.
